# Using the Portapres^®^ for the measurement of toe arterial blood pressure during movement: is it valid and reliable?

**DOI:** 10.14814/phy2.13369

**Published:** 2017-08-07

**Authors:** Joshua A. Goreham, Derek S. Kimmerly, Michel Ladouceur

**Affiliations:** ^1^ Division of Kinesiology School of Health and Human Performance Faculty of Health Professions Dalhousie University Halifax Nova Scotia Canada

**Keywords:** Blood pressure, measurement, movement, validity

## Abstract

The aim of the study was to assess the validity and reliability of using the Portapres^®^ to measure toe blood pressure during rest and exercise. Construct validity, concurrent validity, and interday reliability were assessed by measuring toe (Portapres^®)^) and brachial blood pressure in 16 nondisabled participants on consecutive days. Construct validity was assessed by pedaling on a cycle ergometer (6 revolutions per minute) and comparing the measured toe blood pressure to an estimated value based on orthostatic factors. Concurrent validity was assessed by comparing toe and brachial blood pressure during supine rest and following 10 min of cycling exercise. Interday reliability was assessed by recording toe and brachial blood pressure during supine rest on a second day. Construct validity analysis shows that the toe blood pressure signal was moderately correlated with the changes in heart–toe distance and that changes in toe blood pressure during slow cycling were similar to the estimated value. Resting toe and brachial mean arterial blood pressure showed concurrent validity with only a fixed bias explained by the change in orthostatic pressure and the toe–brachial index. Furthermore, cycling exercise was associated with an increase in brachial and a decrease in toe mean blood pressure. The interday reliability analysis showed no proportional or fixed bias for mean arterial blood pressure. Our study showed the feasibility of using the Portapres^®^ to measure toe blood pressure during movement and can be used to study the effect of movement‐related forces during cycling on toe blood pressure.

## Introduction

There are multiple techniques to measure arterial blood pressure (BP), including the intra‐arterial method, the auscultatory method, the oscillometric method, and the noninvasive continuous (beat‐to‐beat) method (Gerin et al. 2007). Examples of noninvasive type of BP measurement device are the BP monitors from Finapres^®^. Those measurement devices uses the volume‐clamp method, developed by J. Penaz, in association with photoplethysmography (PPG) to measure continuous arterial BP in the finger (i.e., in the common volar digital artery) (de Boer 1985). The cuff maintains vascular volume by using a feedback loop to unload the vascular wall (de Boer 1985) and was developed by Wesseling (1990) and his group in 1982. The Portapres^®^ uses this technology; however, it is portable which allows the patient to be mobile while the device collects accurate and reliable hemodynamic values.

Although the majority of previous studies have measured finger arterial BP using monitors from Finapres^®^, there have only been few published studies that used the device to measure toe arterial blood pressure (TBP) (Kinsella et al. 1990; Rosales‐Velderrain et al. 2011; Hoyer et al. 2013a,b; Quong et al. 2016; Sonter et al. 2015). Kinsella et al. (1990) recognized that the original Finapres^®^ model had not been validated for use on the toe, their results showed that the TBP values followed the changes in finger BP. They concluded that the original Finapres^®^ was an acceptable device for use on the toe. Rosales‐Velderrain et al. (2011) measured TBP using a Finometer^®^ (another model from Finapres^®^). TBP and blood oxygenation were measured in 10 healthy, young volunteers during rest at six different body tilt angles (−6, 0, 10, 30, 70, and 90 degrees). They found a correlation of *r *=* *0.87 (*P *=* *0.01) between the Finometer^®^ and theoretical values based on measured brachial blood pressure combined with calculated orthostatic pressure effect. They found similarities between their TBP measurements at various tilt angles with measurements obtained through instrumented catheters (Katkov and Chestukhin 1980).

Interestingly, Rosales‐Velderrain et al. (2011) stated that the Finometer^®^ accurately measured TBP when compared to theoretical values; however, it has been widely acknowledged that correlation coefficients are not the appropriate statistical analysis to compare two methods of measurement (Ludbrook 1997). Correlation coefficients can only detect random error between two methods of measurements, and therefore systematic error goes undetected. Ordinary least products regression analysis and the Bland–Altman method of differences analysis provide more information on the systematic errors of a device (i.e., fixed and proportional biases) (Ludbrook 1997). Furthermore, Rosales‐Velderrain et al. (2011) experienced several issues while collecting TBP data. First, they were unable to collect TBP data in all participants at 70° and 90° of head‐up tilt. Second, the Finometer^®^ was not built to measure such large BP values that are produced by gravity. Third, Rosales‐Velderrain et al. (2011) mentioned that constriction of the arteries (i.e., vasoconstriction) hampered TBP collection, and that it may have been related to the toes being too cold while at room temperature.

There has been other research that has investigated the validity of PPG for TBP measurements using laser Doppler (LD) (Perez‐Martin et al. 2010; Widmer et al. 2012). Both studies measured TBP in the patients' big toes or in the second toe when the big toe had been amputated. The researchers measured patients' systolic TBPs using the device and a pneumatic cuff as described in Perez‐Martin et al. (2010). They found that the PPG device provided reliable TBP measurements when compared to the LD technique with reported interclass correlation coefficients for the PPG and LD devices, respectively, of 0.887 and 0.893 on the right leg (*n* = 193), and 0.905 and 0.898 on the left leg. The concordance correlation coefficient was 0.913 on the right leg and 0.915 on the left leg, which indicated concordance between the two devices.

These promising TBP measurement results were not reproduced in a second study (Widmer et al. 2012). Using a similar collection protocol, Widmer et al. (2012) found that TBP values varied greatly between PPG and LD devices. The group found nonsignificant mean differences in TBP measurements of 14 mmHg when using a Nicolet VasoGuard (Nicolet Vascular Inc., Madison, WI; PPG1) compared to the Perimed system 5000 (Perimed, Stockholm, Sweden; LD) and Systoe (Atys Medical, France; PPG2) devices. Furthermore, the PPG2 device provided a mean TBP difference of 12 mmHg compared to the LD device. Although the differences in TBPs between devices were not significant, the values did not fall within the acceptable limit of TBP agreement between devices based on their experience. They stated that acceptable limits of agreement would have to be less than 10 mmHg to be sufficient for use in clinical practice. Even though the agreement between the LD and PPG devices was poor, their reported data support that the LD and PPG1 devices had good repeatability. This suggests that the LD and PPG1 devices can be used to collect TBP data; however, switching devices between measurements is not recommended.

Overall, little research on TBP measurements has been conducted, and the existing research shows contradicting results. Due to this reason, the purpose of this study was to further investigate validity (construct and concurrent) and interday reliability of the Portapres^®^ device for the measurement of TBP during rest and slow cadence leg cycling exercise.

## Methods

### Participants

Sixteen nondisabled, normotensive active participants (10 females, 21.9 ± 1.9 years, 1.73 ± 0.1 m, 70.4 ± 9.3 kg) provided informed written consent before beginning the study. Participants were asked to refrain from consuming alcohol, caffeine, nicotine, or performing strenuous bout(s) of physical activity 1 day prior to both experimental days. Participants were well rested (6–8 h of sleep) the night before testing days, consumed their last meal 3 h prior to each testing session, and were well hydrated (i.e., one cup of water per hour) leading up to testing. To minimize diurnal influences on cardiovascular function, participants attended the laboratory at approximately the same time of day for both testing sessions. All participants completed a Physical Activity Readiness Questionnaire (PAR‐Q) (Thomas et al. 1992) prior to testing to ensure they could safely undertake physical exercise. Participants also completed an informed consent form that explained all study protocols and was approved by Dalhousie University's Health Science Research Ethics Board.

### Data collection

The research project used a repeated measures design to answer the three primary research questions. Specifically, TBP was measured during slow cycling at different toe heights and positions (construct validity), resting supine TBP were compared to resting supine brachial BP before and after cycling exercise (concurrent validity), and resting TBP measurements between two testing days (interday reliability).

### Experimental protocol

The study included 2 days of testing for each participant. On the first testing day, resting heart rate (HR) and right brachial BP were measured and recorded manually every minute for 5 min while the participant laid supine. TBP was measured simultaneously from the second toe of the right foot (i.e., from the plantar digital artery). The Portapres^®^ front‐end unit was attached just proximal and anterior to the participants' right ankle.

On the second testing day, participants completed an initial 5‐min resting condition in which HR, right brachial BP, and right TBP were measured. Participants then sat on an electromagnetically braked cycle ergometer (Velotron^®^, RacerMate Inc., Seattle, WA) with the seat adjusted to 100% of their right greater trochanter height (Nordeen‐Snyder 1977). The seat height was based on greater trochanter height to keep limb segment angles similar between participants.

Starting with their foot at the top of the pedal crank, the participant pedaled at six revolutions per minute for 1 min to determine if the change in TBP corresponded to the change in toe height. The frequency of pedaling was controlled through auditory feedback from a metronome set at 60 beats per minute and the participant was required to be back at the top of the pedal crank every 10 beats. This condition was called the “slow orthostatic” trial, and was used to measure construct validity. An infrared marker was attached to the fifth metatarsal of the right foot. The right toe position was measured using an Optotrak Certus^®^ camera system (Northern Digital Inc., Waterloo, Canada).

Following the completion of the slow orthostatic trial, participants immediately completed 10 min of cycling exercise at a cadence of 50 revolutions per minute and a mechanical power output that elicited 60% of their age‐predicted maximum heart rate (HR_max_) (Tanaka et al. 2001). Immediately following the bout of cycling exercise, the participant dismounted from the cycle ergometer and rested supine. Heart rate, brachial BP, and TBP were measured for 5 min starting when the participant was in a supine position. Following the 5 min measurement period, the participant continued to rest until their brachial BP and HR returned to pre‐exercise values, and then they were allowed to leave the laboratory. The time between the end of the exercise bout and the first BP measurement was recorded to ensure all participants BP measurements were collected at similar times after exercise.

### Outcome measures

Heart rates, brachial BP, and TBP were measured using a Polar FT1 HR monitor (Tempe Oy, Finland), a Carescape v100 blood pressure monitor (General Electric Healthcare, Mississauga, Canada), and a Portapres^®^ Model‐2 blood pressure monitor (Finapres^®^ Medical Systems, Amsterdam, the Netherlands), respectively. Heart rate was measured to ensure participants cycled at an intensity that elicited 60% of their HR_max_.

The three‐dimensional position of the fifth metatarsal and the output signal of the Portapres^®^ were sampled at a frequency of 200 Hz and analyzed using NDI First Principles^™^ software and custom MATLAB scripts.

### Data analysis

#### Construct validity

TBP data from the “slow orthostatic” trial were filtered at 0.3 Hz using a fourth‐order low‐pass Butterworth filter to subtract the systolic pulse from the measurement. The actual change in TBP due to corresponding fluctuations in orthostatic pressure was measured by calculating the difference between maximum and minimum TBP values for each pedal revolution during slow cycling. The theoretical change in TBP during slow cycling was determined by the multiplication of blood density (1053 kg/m^3^) (Kenner 1989), acceleration due to gravity (9.81 m/sec^2^), and diameter of pedal crank (0.36 m). The hypothetical value was equal to 27.9 mmHg.

The fifth metatarsal position data were filtered at 1 Hz using a fourth‐order low‐pass Butterworth filter to monitor pedal position and timing of the pedal revolution. The vertical changes in position of the fifth metatarsal (i.e., toe height) were extracted from the kinematic dataset and synchronized with the TBP data.

#### Concurrent validity

Concurrent validity was assessed by comparing toe mean arterial pressure (MAP_T_) and brachial mean arterial pressure (MAP_B_) measurements at two different parts of the experimental protocol: (1) during the initial rest period on the second testing day and (2) immediately following the cycling exercise bout. For this analysis, the TBP data were filtered using a 5‐Hz fourth‐order low‐pass Butterworth filter for all participants' data. The filtered TBP data were then extracted 15 sec before and 15 sec after the fourth minute manual brachial BP measurement. Maximum and minimum TBP values were classified as systolic TBP and diastolic TBP values, respectively. MAP_T_ and MAP_B_ were then calculated using systolic and diastolic TBP and brachial BP values measured at the fourth minute of rest using Equation (1) below. The same analysis protocol was used for the postexercise analysis.

(1) MAP=Diastolic+[1/3(Systolic ‐ Diastolic)]

#### Interday reliability

MAP_T_ and MAP_B_ measurements from the initial resting periods on both testing days were analyzed to determine interday reliability. The same analysis procedure used for the concurrent validity portion of the study was used to determine MAP_T_ and MAP_B_ for interday reliability.

### Statistical analysis

#### Construct validity

A one‐sample *t*‐test was used to compare each change in TBP during slow cycling (6 rpm) for all participants to the theoretical value of 27.9 mmHg. Six TBP values were measured and compared against the theoretical value for each participant, which corresponded to the six full revolutions of the pedal crank. The D'Agostino and Pearson normality test was applied to all individual data points to ensure the data were normally distributed. Statistical significance determination was set at *P* < 0.05. A cross‐correlation analysis was used to assess the similarity between the participant's filtered kinematic and TBP waveforms.

#### Concurrent validity

Concurrent validity was assessed by contrasting the brachial BP and TBP measurements at rest and following a bout of cycling exercise. The brachial BP and TBP at rest were analyzed using two different statistical methods: a model II ordinary least products (OLP) regression analysis and a method of differences (Altman and Bland 1983). Concurrent validity related to the effect of exercise included an OLP regression analysis on the change in MAP_T_ (∆MAP_T_; *x*‐axis) versus the change in MAP_B_ (∆MAP_B_; *y*‐axis) data. The changes in MAP were calculated by subtracting the MAP after exercise from the MAP before exercise for both the toe and brachial measurement sites.

The OLP regression analysis assessed the fixed and proportional bias between both measurement sites (brachial and toe), and was chosen because it accounts for random error within both sets of measurements (Ludbrook 1997). For the OLP regression analysis, MAP, systolic, and diastolic BP values for the toe and brachial measurement sites were plotted on the *x*‐axis and *y*‐axis, respectively. The OLP analysis calculated the *y* intercept (*a*′) and the slope (*b*′) of the OLP regression line to determine biases. Fixed bias was determined by calculating the 95% confidence intervals (CI) for *a*′ and determining whether it included the value of “0”. If the 95% CI band did not include “0”, the data were classified as having a fixed bias. Proportional bias was defined as “one measurement that produces values that are greater (or lower) than those from the other by an amount that is proportional to the level of the measured variable” (Ludbrook 1997). Therefore, proportional bias existed if the 95% CI for *b*′ did not include a value of “1”.

In the method of differences (Altman and Bland 1983), the differences between MAP_B_ and MAP_T_ (*y*‐axis) were plotted against the average of the two MAP measures (*x*‐axis). Proportional bias was determined by applying an ordinary least squares (OLS) regression to the Bland–Altman method of differences data. A one‐sample *t*‐test comparing a value of “0” to the slope of the method of difference data (OLS regression line) was used to determine proportional bias. Separately, a one‐sample *t*‐test comparing the mean difference data to a value of “0” was used to determine fixed bias.

The D'Agostino and Pearson normality test was applied to all individual data points to ensure that data were normally distributed (D'agostino 1986). Statistical significance was set at *P* < 0.05 (with a Bonferroni correction for multiple comparisons).

#### Interday reliability

All MAP, systolic, and diastolic TBP measurements acquired on two consecutive days were tested for interday reliability using OLP and Bland–Altman analyses. These OLP and Bland–Altman analyses used the same method as the concurrent validity analysis. For the OLP regression analysis, MAP, systolic, and diastolic TBP values for the first and second days were plotted on the *x*‐axis and *y*‐axis, respectively. For the Bland–Altman method of differences analysis, the interday average was plotted on the *x*‐axis, and the difference between the days value were plotted on the *y*‐axis. Proportional and fixed biases for both statistical tests also used the same method as presented in the “Concurrent validity” analysis section above. The D'agostino and Pearson normality test was applied to all individual data points to ensure the data were normally distributed (D'agostino 1986). Statistical significance was set at *P *< 0.05 (with a Bonferroni correction for multiple comparisons). The OLS regression line was not normally distributed and was analyzed using the Wilcoxon signed‐rank test.

## Results

### Construct validity

**Figure 1:**
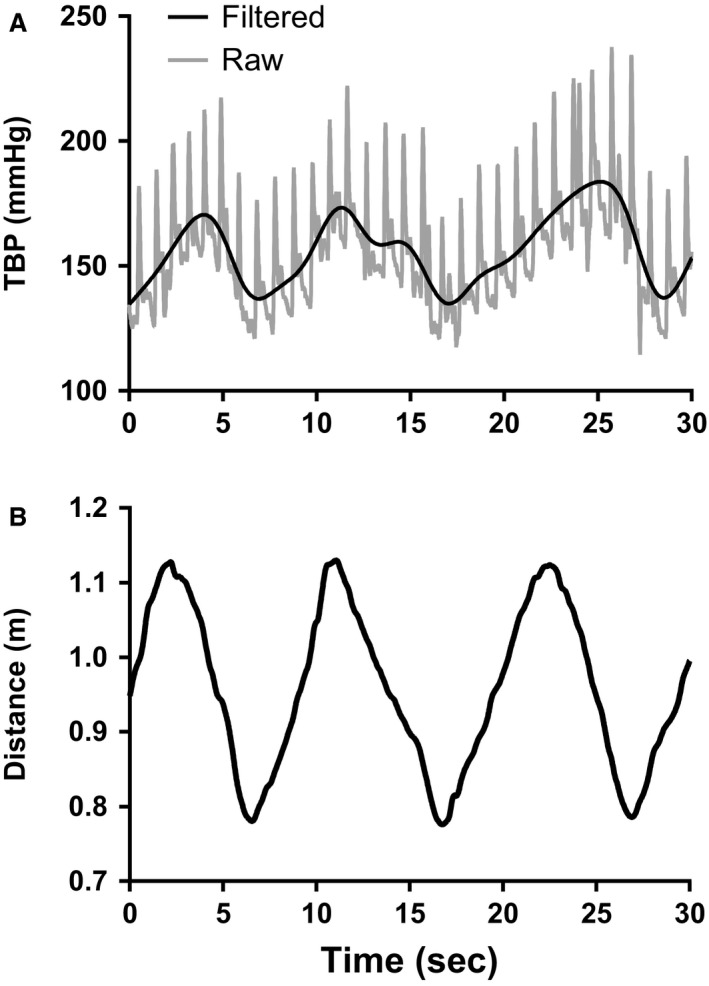
Example of TBP during slow cycling for one participant. (A) Raw and filtered TBP data in mmHg and (B) distance between heart and toe in meters. mmHg, millimeters of mercury; m, meters; TBP, toe blood pressure.

An example of one participant's raw and filtered TBP (Fig. 1A) as well as synchronized distance between the heart and the toe (Fig. 1B) during a portion of their slow orthostatic trial is shown in Figure 1. This figure demonstrates that fluctuations in TBP follow corresponding changes in the distance between the heart and toe, which is related to differences in orthostatic pressure.

Fifteen participants provided 76 acceptable TBP values out of a maximum of 96 possible measurement, which were included in the construct validity analysis. One participant's data were excluded because TBP could not be measured. From the remaining 90 TBP values, 14 TBP data points were either absent or were unable to be extracted from the raw data due to participant error (i.e., not cycling at the appropriate cadence, applying too much pressure on the TBP cuff, not completing all of the revolutions). Only one TBP value was deemed an outlier (75.0 mmHg) and was excluded from analysis due to being more than 3 SD from all participants overall mean (mean + 3 SD = 61.3 mmHg).

The cross‐correlation analysis (*n* = 7) between the filtered TBP signal and difference in height between the heart and toe signal showed a moderate correlation (*r* = 0.66 ± 0.16) with no difference in the timing (lag = 0 ± 0.86 sec) between the two signals. Furthermore, the mean difference in TBP (28.7 ± 10.9 mmHg; 95% CI's = 26.3–31.2) was similar to the proposed theoretical difference of 27.9 mmHg (*t* = 0.682, df = 75, *P *<* *0.497).

### Concurrent validity

**Figure 2:**
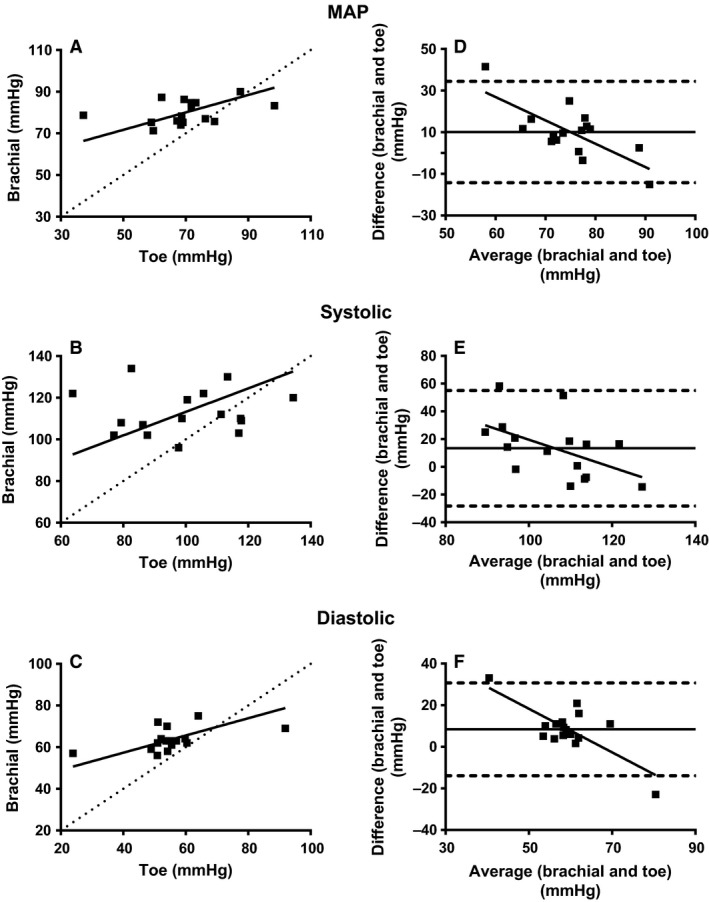
Concurrent validity plots of TBP at rest using OLP regression and Bland–Altman method of differences analyses. OLP analysis for TBP are presented for MAP (A), systolic pressure (B), and diastolic pressure (C). The OLP line of best fit is represented with a solid line. A dotted line represents the line of unity. The Bland–Altman method of differences analysis of TBP is presented for MAP (D), systolic pressure (E), and diastolic pressure (F). The mean difference between measurement sites and 95% limits of agreement are represented, respectively, with a solid line and dotted lines. The OLS line of best fit is represented by the diagonal solid line. mmHg, millimeters of mercury; OLS, ordinary least products.

Concurrent validity was explored by determining whether MAP_T_ and MAP_B_ values were related during rest, and following a cycling exercise bout. The average MAP_T_ during rest for all 16 participants was 69.9 ± 13.2 mmHg (systolic: 99.4 ± 18.9 mmHg; diastolic: 55.2 ± 13.1 mmHg) and average MAP_B_ was 80 ± 5.5 mmHg (systolic: 112.9 ± 10.7 mmHg; diastolic: 63.6 ± 5.4 mmHg). Ordinary least products (OLP) analysis indicated that there was fixed bias, but no proportional bias in MAP between the two measurement sites (Table 1; Fig. 2A) at rest. The OLP analysis also showed that there were no proportional or fixed bias in systolic BP, but there was proportional and fixed bias in diastolic BP between measurement sites (Table 1, Fig. 2C). The Bland–Altman method of difference analysis shows a presence of fixed bias and proportional bias in MAP, and diastolic BP between both measurement sites during rest (Table 1, Figs. 2D and F). In agreement with the OLP analysis, the Bland–Altman method of difference analysis did not show fixed or proportional bias in systolic BP (Table 1, Fig. 2B and E). The only measurement in which the OLP and Bland–Altman analyses did not agree was the proportional bias for MAP. For this comparison, the Bland–Altman analysis showed a proportional bias, whereas the OLP analysis did not.

**Table 1:** Concurrent validity outcomes between brachial and toe BP at rest using the Bland–Altman method of differences and the ordinary least products (OLP) regression analyses

Variable	Bland–Altman results				OLP results			
	*r*	*b*	*P* (OLS)	Proportional bias	*r*	*b*′	95% CI for *b*′	Proportional bias
MAP	0.73	−1.12	0.001	Yes	0.36	0.42	−0.21, 1.05	No
Systolic	0.52	−0.99	0.04	Yes	0.05	0.56	−6.31, 7.43	No
Diastolic	0.75	−1.05	0.0007	Yes	0.50	0.41	0.002, 0.83	Yes
	**Mean difference ± SEM**	**95% CI for mean difference**	***P* (*t*‐test)**	**Fixed bias**		***a*′**	**95% CI for *a*′**	**Fixed bias**
MAP	10.09 ± 3.1	3.5, 16.7	0.005	Yes		50.8	6.1, 95.5	Yes
Systolic	13.44 ± 5.3	2.1, 24.8	0.02	Yes		56.8	−637.7, 751.3	No
Diastolic	8.43 ± 2.8	2.4, 14.5	0.01	Yes		40.8	17.5, 64.1	Yes

*r*, product–moment correlation coefficient; *a*′, *b*′ coefficients in ordinary least products regression model *Y* = *a*′ + *b*′ (*X*). For Bland–Altman method of differences analyses: *b*, ordinary least squares (OLS) slope of the Bland–Altman method of differences plots; *P* (OLS), the *P* value for the OLS slope (vs. 0); CI, confidence interval; *P* (*t*‐test), the *P* value for the one‐sample *t*‐test on the mean differences (vs. 0); *P* < 0.05. For OLP analyses: *α*′, *y* intercept; *b*′, slope; proportional bias, if 95% CI for *b*′ does not include 1; fixed bias, if 95% CI for *α*′ does not include 0.

**Figure 3:**
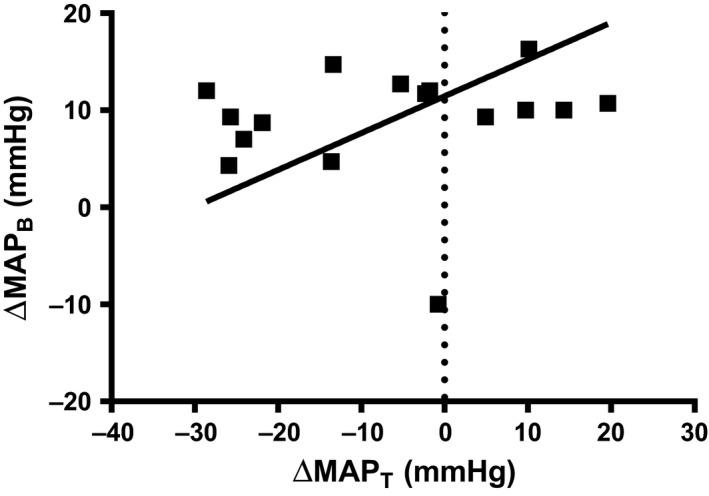
The effect of exercise on the changes in MAP in the toe (ΔMAP_T_) and brachial (ΔMAP_B_) arteries. OLP analysis of ΔMAP_T_ and ΔMAP_B_ between rest and after exercise is presented in the figure. The OLP line of best fit is represented with a solid line. The vertical dotted line represents no differences (zero‐line). mmHg, millimeters of mercury; OLP, ordinary least products.

The second method to determine the concurrent validity of TBP was to compare the effect of exercise on both MAP_T_ and MAP_B_. The average elapsed time after exercise to obtain resting TBP and brachial BP measurements was 28.9 ± 13.9 sec. The average MAP_T_ immediately after cycling exercise was 62.5 ± 11.9 mmHg (systolic: 82.7 ± 19.7 mmHg; diastolic: 52.4 ± 11.6 mmHg) and average MAP_B_ was 89 ± 7.2 mmHg (systolic: 135.3 ± 12.5 mmHg; diastolic: 65.9 ± 6.7 mmHg). The OLP analysis that was applied to the change in MAP_T_ data versus the change in MAP_B_ data showed a slope (*b*′) of 0.38 (95% CI's: −1.3 to 2.1), and a *y* intercept (*a*′) of 11.5 (95% CIs: −17.1 to 40.0) (Fig. 3). The 95% CIs for *b*′ and *a*′ showed that there were no proportional or fixed biases in the data when comparing the change in MAP_T_ and MAP_B_ due to cycling exercise. MAP_B_ increased in 15 of 16 participants after exercise, whereas MAP_T_ increased in 5 participants and decreased in 11 participants after the cycling exercise condition.

### Interday reliability

**Figure 4:**
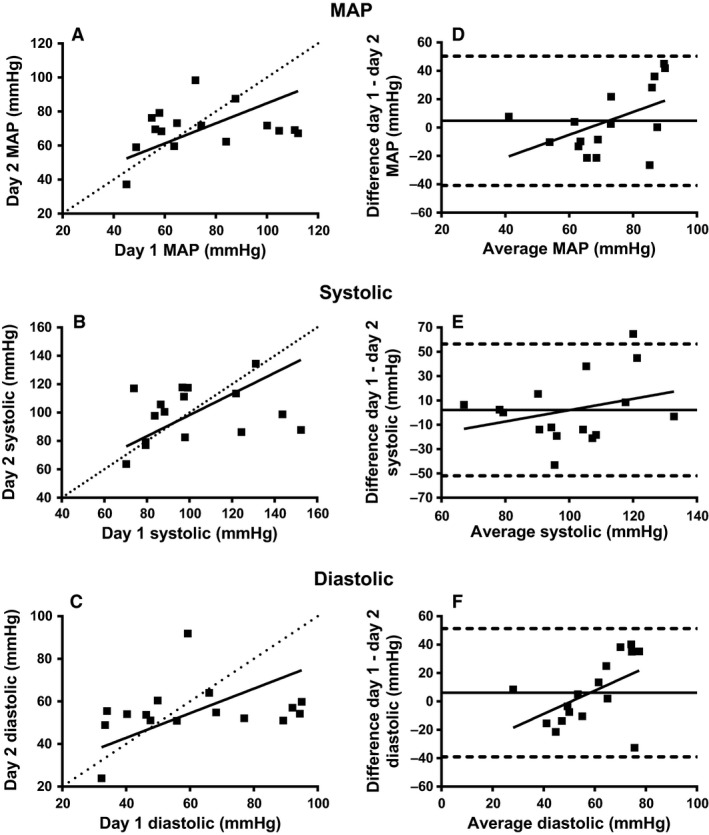
Interday reliability plots of TBP using OLP regression and Bland–Altman method of differences analyses. OLP analysis for TBP presented for MAP (A), systolic pressure (B), and diastolic pressure (C). The OLP line of best fit is represented with a solid line. A dotted line represents the line of unity. The Bland–Altman method of differences analysis of TBP is presented for MAP (D), systolic pressure (E), and diastolic pressure (F). The mean difference between days and 95% limits of agreement are represented, respectively, with a solid line and dotted lines. The OLS line of best fit is represented by the diagonal solid line. mmHg, millimeters of mercury; OLP, ordinary least products.

Interday reliability validity was tested by measuring supine TBP at rest on two consecutive day. The average MAP_T_ during rest for all 16 participants on day 1 was 74.8 ± 22.5 mmHg (systolic: 101.6 ± 25.4 mmHg; diastolic: 61.3 ± 22.6 mmHg), whereas on day 2 it was 69.9 ± 13.2 mmHg (systolic: 99.4 ± 18.9 mmHg; diastolic: 55.2 ± 13.1 mmHg). An OLP analysis showed that there were no fixed or proportional bias in MAP, systolic, or diastolic measurements in the toe between the two testing days (Table 2; Figs. 4A–C). The Bland–Altman method of differences analysis also demonstrated no fixed bias or proportional bias in MAP, and systolic BP between the 2 days (Table 2, Fig. 4D–E). For the diastolic TBP, there was a proportional bias (*P *<* *0.05) but no fixed bias (Table 2; Fig. 4F).

**Table 2:** Interday reliability outcomes of different quantifiable measures of BP by the Bland–Altman method of differences and the ordinary least products (OLP) regression analyses

Variable	Bland–Altman results				OLP results			
	*r*	*b*	*P* (OLS)	Proportional bias	*r*	*b*′	95% CI for *b*′	Proportional bias
MAP	0.50	0.80	0.05	No	0.23	0.59	−0.814, 1.994	No
Systolic	0.30	0.47	0.26	No	0.25	0.74	−0.935, 2.422	No
Diastolic	0.51	0.82	0.04	Yes	0.25	0.58	−0.689, 1.844	No
	**Mean difference ± SEM**	**95% CI for mean difference**	***P* (*t*‐test)**	**Fixed bias**		***a*′**	**95% CI for *a*′**	**Fixed bias**
MAP	4.81 ± 5.8	−7.6, 17.2	0.42	No		25.87	−83.43, 135.2	No
Systolic	2.21 ± 6.9	−12.5, 17.0	0.75	No		23.86	−151.7, 199.4	No
Diastolic	6.11 ± 5.8	−6.2, 18.4	0.40	No		19.81	−62.62, 102.2	No

*r*, product–moment correlation coefficient; α′, *b*′ coefficients in ordinary least products regression model *Y* = a′ + b′ (*X*); For Bland–Altman method of differences analyses: *b*, ordinary least squares (OLS) slope of the Bland–Altman method of differences plots; *P* (OLS), the *P* value for the OLS slope (vs. 0); CI, confidence interval; *P* (*t*‐test), the *P* value for the one‐sample *t*‐test on the mean differences (vs. 0); *P* < 0.05. For OLP analyses: *a*′, *y* intercept; *b*′, slope; proportional bias, if 95% CI for *b*′ does not include 1; fixed bias, if 95% CI for *a*′ does not include 0.

## Discussion

The aim of this study was to determine the validity and reliability properties of the Portapres^®^ BP monitor for measuring TBP in nondisabled individuals. Three experiments were completed using a Portapres^®^ BP monitor in attempt to answer the primary research question. First, construct validity was determined by comparing the actual change in measured TBP to an expected change in TBP during slow cycling. Theoretically speaking, it was believed that the changes in TBP that occurred were due to the change in the orthostatic component (i.e., gravity) within each revolution during cycling. Second, the concurrent validity of the Portapres^®^ was determined by comparing the measured TBP to the brachial BP at rest and after a bout of cycling exercise. Third, the interday reliability of the Portapres^®^ was determined by comparing resting TBP values on the first testing day to resting TBP values on the second testing day. The study was unique because it was the first one to use the Portapres^®^ during any type of lower body movement. The study provided interesting findings, which adds to the literature as only a few other published studies have used a similar BP recording device on the toe while at rest (Kinsella et al. 1990; Rosales‐Velderrain et al. 2011; Hoyer et al. 2013a,b; Quong et al. 2016; Sonter et al. 2015). Second, this study is the first one to use a thorough battery of statistical validity test to assess the validity of using the Portapres^®^ to collect BP data on the toe. Finally the results from this study will be of interest to researcher studying the regulation of arterial blood pressure in the lower limb, as well as, clinical researchers involved with peripheral artery disease and its rehabilitation.

### Construct validity

The changes in TBP were due to the change in the toe distance from the heart during slow cycling (i.e., an orthostatic effect); however, they included some variability. Our results show that the TBP measured by the Portapres^®^ was moderately correlated, with an appropriate magnitude, to the change in height between the heart and toe while cycling at a slow cadence.

Discrepancies between theoretical values and our results were expected as human physiology can rely on multiple variables (i.e., level of hydration, vascular resistance, tissue temperature, consistency of movement and movement speed) that are not present in controlled environments. In a previous study, Rosales‐Velderrain et al. (2011) compared the theoretical differences in TBP to the actual measured TBP and reported a correlation value (*r *=* *0.87), which is higher than the one reported in this study (*r *=* *0.66). Their measurements were collected under static conditions, and apparent differences in theoretical and actual measurements were found; however, these differences were not quantified. The difference between the Rosales‐Velderrain et al. (2011) and this study on TBP measurements are not completely understood; however, some suggestions can be made. TBP in this study was expected to oscillate with a change between the heart and toe level of 0.36 m during slow cycling. A kinematic analysis showed an average change in fifth metatarsal height of 0.338 ± 0.011 m, which is smaller than the value used for the calculation of the theoretical change in TBP (−0.022 m). Using the fifth metatarsal change in height instead of the crank diameter would yield a smaller theoretical value for the change in TBP (26.2 mmHg). As mentioned in the Results section, the 95% CIs of the change in TBP were 26.3–31.2, meaning the majority of TBP values were above 26.2 mmHg.

Second, the participants were instructed to keep a slow and steady cadence of six revolutions per minute during the 1 min slow orthostatic trial. At times, participants may have generated excessive forces on the pedal crank, which could have caused an increase in cuff pressure at the toe. If this was true, then larger pressures would be expected. Although pedal force was not measured in this study, the toe position versus time graphs showed very little deviation from the expected toe position curve in participants (Fig. 1). As mentioned previously, if the researcher noticed the participant was cycling too fast (or too slow), then the corresponding TBP for that revolution was not used in the analysis.

Pulse pressure and difference between the systolic (maximum) and diastolic (minimum) blood pressures in one heart contraction may have also played a role in the changes of TBP compared to the change in foot height. One noticeable difference in the raw TBP waveforms was that they were not comparable between participants. Some participants showed very large and pronounced pulse pressures, whereas others showed very small pulse pressures. This difference between participants could be the result of many factors related to the alignment of the cuff or the toe temperature. However, the small difference in measurement magnitude between two consecutive days (MAP: 4.81 mmHg, Table 2) provides some evidence of the robustness of the interparticipant differences.

With a measured change in TBP of 28.7 mmHg in comparison to the theoretical estimated value of 27.9 mmHg, this study shows that the Portapres^®^ may have some minimal measurement error (~2 mmHg) when measuring TBP during a lower limb movement like cycling. One of the goals with slow cycling at six revolutions per minute was to observe any changes in TBP that were due to systolic and orthostatic forces. The systolic force was mostly attenuated from the raw TBP waveform by filtering, and was therefore believed to have very little effect on the TBP measurements during slow cycling. That said, it was difficult to predict how much of an effect the slow cycling movement had on the measured TBP values. Future studies should investigate ways to control for movement artifact when measuring the effect of the orthostatic force on TBP.

### Concurrent validity

To test the concurrent validity of the Portapres^®^ to measure TBP it was important to compare the measurements against another device and measurement site, as well as comparing the TBP values collected in this study to TBP values collected by others to determine their normalcy. The results of our study show that brachial MAP was approximately 10 mmHg (10.09 ± 3.1, Table 2) greater than toe MAP. However, this difference was mostly dependent on the toe MAP (proportional bias, Table 1). These results are congruent to the ones presented by Sahli et al. (2004), which reported a decrease of approximately 10 mmHg between brachial BP and TBP.

**Figure 5:**
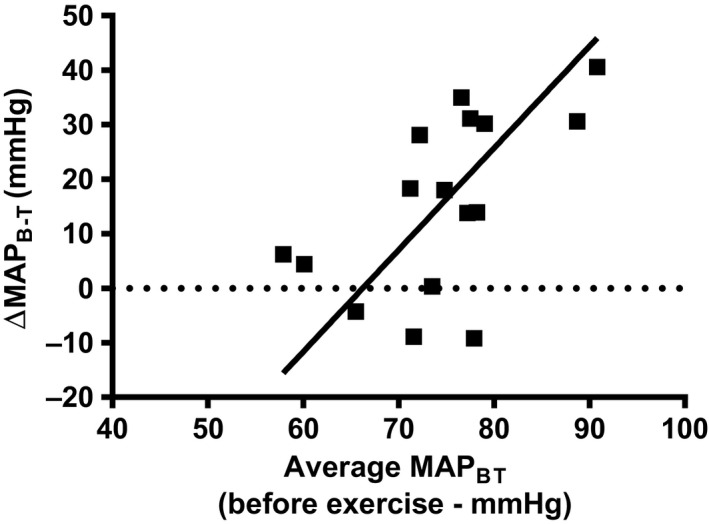
The change in brachial and toe MAP after exercise compared to rest (ΔMAP_B‐T_) versus the average resting MAP (MAP_BT_). The OLP line of best fit is represented with a solid line. mmHg, millimeters of mercury.

Another notable finding of the current study was how the difference in MAP_T_ and MAP_B_ changed after exercise in relation to the sum of the MAP for the same two measurement sites at rest (see Fig. 5). A trend occurred and showed that the difference in MAP between the brachial and toe measurement sites increased more after exercise in participants who had greater total resting MAP (i.e., an average of brachial and toe MAP). An OLP analysis was also applied to the data in Figure 5, and a slope (*b*ʹ) of −1.87 and a *y* intercept (*a*ʹ) of −123.8 were calculated. The 95% CI for the *b*ʹ was −0.4 to 3.4, meaning there was no proportional bias, and the 95% CI for *a*ʹ was −235.5 to −12.0, meaning there was a fixed bias present in the data. Although the mechanisms behind this finding are not currently understood, it gives preliminary evidence that differences between upper and lower body measurement sites become larger when there is a greater average resting MAP between the two measurement sites.

### Interday reliability

The results of this study show that TBP measured using the Portapres^®^ had very good interday reliability with only the Bland–Altman analysis showing a proportional bias for diastolic measurements. However, the limits of agreement in the TBP measurements were greater than the acceptable limit of agreement of 10 mmHg that has been suggested for clinical practice (Widmer et al. 2012). The limits of agreement in this study were much greater than 10 mmHg (i.e., −40 to 50 mmHg). These limits of agreement are comparable to the ones reported by De Graaff et al. (2000) using LD, and PPG techniques in 60 patients that were at risk for vascular disease. The group filtered LD data using two different methods (LD_3_: 3 sec; LD_0.03_: 0.03 sec) to detect changes in blood flow (LD_3_) and heart beats (LD_0.03_). Their results showed limits of agreement that were well above 10 mmHg as well (LD_3_: −27 to 34; LD_0.03_: −24 to 34; and PPG: −29 to 36), and were comparable to results of the current study because of the lower TBP magnitude found in their at‐risk population (approximately 60 mmHg vs. 72 mmHg in this study).

The significance of these findings is that the Portapres^®^ cannot, at this time, be used to measure TBP in a clinical population until it can provide lower limits of agreement between measurements on separate days (i.e., better interday validity). For example, according to the TBP values found using our findings, a group of patients with peripheral artery disease taking part in an exercise intervention study over an extended period would have to provide very large changes in TBP (i.e., more than 45 mmHg) to show any sign of actual benefit from the exercise. The large amount of change in TBP is probably not feasible in a disabled population considering they have very low TBP to begin with. Therefore, it would not be appropriate to expect such large changes in this population due to exercise. Future studies should investigate other measurement protocols to increase interday reliability with the Portapres^®^ in attempt to lower the limits of agreement, so peripheral artery disease detection and rehabilitation progress can be measured using the device.

In conclusion, we have shown that the Portapres^®^ was able to collect TBP in 16 nondisabled participants at rest and while cycling at a very slow cadence with mixed results regarding construct and concurrent validity as well as interday reliability. Construct validity has been established through the moderate cross‐correlation and similar magnitude of changes between the measured TBP and the changes in height between the heart and the toes during slow cycling. We found differences in concurrent validity depending on whether the measurements were recorded at rest or after cycling exercise. Brachial BP and TBP had fixed but no proportional bias at rest prior to exercise, which is a promising result. This result was not found after exercise which is believed to be due to different regional effect in vascular resistance. Finally, the Portapres^®^ did not show acceptable limits of agreement when testing for interday reliability (i.e., <10 mmHg, or as small as brachial BP limits of agreement), but our findings do provide information to determine sample size calculations in future studies. Finally, the results from this study show the possibility of using the Portapres^®^ to study the effect of movement‐related increases in arterial blood pressure (Sheriff et al. 2009) during lower limb activities like cycling or walking in able‐bodied participants and participants with cardiovascular impairment like peripheral artery disease or diabetes.

## Conflict of Interest

None declared.
